# Atherosclerotic Plaque Tissue Characterization: An OCT-Based Machine Learning Algorithm With *ex vivo* Validation

**DOI:** 10.3389/fbioe.2020.00749

**Published:** 2020-07-02

**Authors:** Chunliu He, Zhonglin Li, Jiaqiu Wang, Yuxiang Huang, Yifan Yin, Zhiyong Li

**Affiliations:** ^1^School of Biological Science and Medical Engineering, Southeast University, Nanjing, China; ^2^Department of Neurosurgery, The Affiliated Hospital of Xuzhou Medical College, Xuzhou, China; ^3^School of Mechanical, Medical, Process Engineering, Queensland University of Technology, Brisbane, QLD, Australia

**Keywords:** atherosclerotic plaque, carotid artery, histology, machine learning, optical coherence tomography

## Abstract

There is a need to develop a validated algorithm for plaque characterization which can help to facilitate the standardization of optical coherence tomography (OCT) image interpretation of plaque morphology, and improve the efficiency and accuracy in the application of OCT imaging for the quantitative assessment of plaque vulnerability. In this study, a machine learning algorithm was implemented for characterization of atherosclerotic plaque components by intravascular OCT using *ex vivo* carotid plaque tissue samples. A total of 31 patients underwent carotid endarterectomy and the *ex vivo* carotid plaques were imaged with OCT. Optical parameter, texture features and relative position of pixels were extracted within the region of interest and then used to quantify the tissue characterization of plaque components. The potential of individual and combined feature set to discriminate tissue components was quantified using sensitivity, specificity, accuracy. The results show there was a lower classification accuracy in the calcified tissue than the fibrous tissue and lipid tissue. The pixel-wise classification accuracy obtained by the developed method, to characterize the fibrous, calcified and lipid tissue by comparing with histology, were 80.0, 62.0, and 83.1, respectively. The developed algorithm was capable of characterizing plaque components with an excellent accuracy using the combined feature set.

## Introduction

Rupture of vulnerable atherosclerotic plaques is the leading cause of stroke and myocardial infarction (Cicha et al., 2011). These serious accidents often occur when plaques in the arteries suddenly rupture, causing thrombus and leading to the obstruction of the blood flow to the brain or the heart (Lekadir et al., 2017). Therefore, early and accurate prediction of individuals at high risk of plaque rupture could allow preventive, therapeutic, or surgical interventions to be taken to prevent such life-threatening events happening.

It is now well established that plaque components and morphology are the main factors in the determination of plaque vulnerability (Shah, 2003; Li et al., 2006a, b). Plaques with a large lipid core and a thin fibrous cap are more prone to rupture, whereas plaques containing calcified tissue may tend to be more stable (Arbab-Zadeh and Fuster, 2015). High-resolution intravascular optical coherence tomography (OCT) imaging has shown great promise in the identification and characterization of atherosclerotic plaque components, such as fibrous cap, calcification and lipid tissue, as well as the quantification of plaque areas and volume (Yabushita et al., 2002; Regar et al., 2013). Histological studies have shown the ability of OCT to separate fibrous, calcified and lipid tissue from the carotid artery (Zimarino et al., 2007; Matsumoto et al., 2014). Although OCT images present plaque morphological information with a relatively high resolution, it still relies on interpretation of the images by trained readers for the identification and quantitation of plaque components. Therefore, development of computational techniques is important to determine plaque components.

Levitz et al. (2004) published a quantitative study which demonstrated that OCT tissue characterization of atherosclerotic plaques could be conducted by measuring by the backscattering and attenuation coefficients. Their work showed that the coefficients has essential differences between fibrous, lipid and calcific plaques. The optical parameters were gradually enriched for atherosclerotic characterization in OCT quantitative studies (van der Meer et al., 2005a, b; Xu et al., 2008; Popescu et al., 2010; van Soest et al., 2010). However, tissue quantification using only optical parameters caused significant overlaps between different tissue types. In addition, discrepancies existed because of the different light sources and physical models. Later, Wang et al. (2010) proposed a different methodology using the morphology operation for semiautomatic segmentation of calcified plaques in OCT images. Then, a series of such studies were implemented to quantify plaque components, by combining optical parameters and texture features (Ughi et al., 2013), k means and texture features (Athanasiou et al., 2014), and least square optimization strategy to estimate the depth profiles in OCT data (Rico-Jimenez et al., 2016). Previous studies demonstrate the feasibility of atherosclerotic plaques segmentation based on machine learning algorithm, while the segmentation results were compared with manual annotation. Recently, the prevailing convolutional neural networks (CNN) were also applied to the classification of plaque components based on OCT images and demonstrated excellent results (Abdolmanafi et al., 2017, 2018; Gessert et al., 2019). However, it is still challenging to segment plaque components based on OCT imaging. Moreover, lack of *ex vivo* validation on the developed imaging processing methods is the other obstacle in this area.

This study was designed to characterize and identify the fibrous, calcified and lipid tissues based on the expert annotation using histology images. In the present study, we used optical parameters, texture features combined with relative position of pixels to analyze and delineate plaque components in OCT images. Validation algorithm was performed by comparing these results with those of corresponding histological sections of the *ex vivo* carotid plaques.

## Materials and Methods

### Carotid Plaque Tissue Collection

In this study, 31 patients with a high-grade (>70%) carotid stenosis scheduled for carotid endarterectomy (CEA) from October 2015 to December 2018 were included. The dissected specimens were used for both *ex vivo* OCT imaging and histology analysis. All participants provided a written informed consent prior to the enrollment and the study protocol was approved by the institutional ethics committee.

### OCT Image Protocol and Preprocessing

**FIGURE 1:**
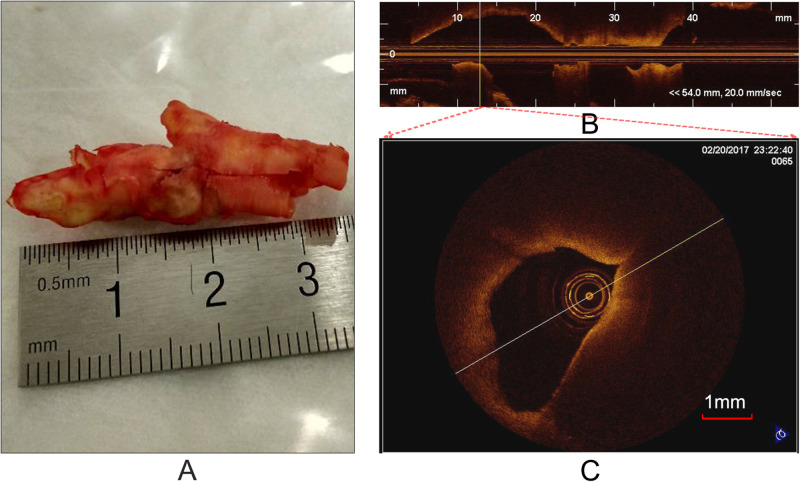
Carotid plaque tissue and OCT imaging. **(A)** An intact CEA plaque tissue. **(B)** A longitudinal view of CEA tissue **(A)** with the C7-XR OCT system. **(C)** A cross-sectional OCT image from the vertical line **(B)**.

OCT can acquire cross-sectional images (Figure 1C) of microscopic pathology structure of arterial wall. In the study, the OCT system used for the CEA plaque tissue imaging was a commercially available C7-XR with Dragonfly^TM^ catheter (2.7 F, C7-XR, St. Jude Medical Inc., St. Paul, MN, United States). The OCT system had high image resolutions of ∼15 μm, approximately 10 times finer than conventional intravascular ultrasound. Scan parameters were set as 100 frames/s, 54,000 A-scans/s, pullback speed of 20 mm/s, pullback length of ∼54.2 mm (Figure 1B). In average, the *ex vivo* specimen was imaged over 50–250 frames, depending upon the actual length of the samples.

Prior to OCT imaging, the specimens (Figure 1A) were flushed with phosphate buffered saline (PBS) to remove blood clots, washing out excess blood to reduce possible clutter artifacts. In order to accurately co-register the OCT images with the histological and immunohistochemistry sections, at each imaging site, photograph was taken and stored to eliminate the rotary bias on slices as much as possible. In each imaging site, an automatic pullback was performed to obtain images. Cross-sectional images (cartesian coordinate images) and lossless raw images (polar coordinate images) were acquired and stored on CD-ROMs for off-line process. It is worth noting that the raw images were used as input and the cross-sectional images were used as visualization.

Lumen segmentation is the primary step for plaque image analysis. The lumen boundary is solved by the classic optimization method, dynamic programming (DP) (Amini et al., 1990). More details about the algorithm refer to literature (Wang et al., 2012).

**FIGURE 2:**
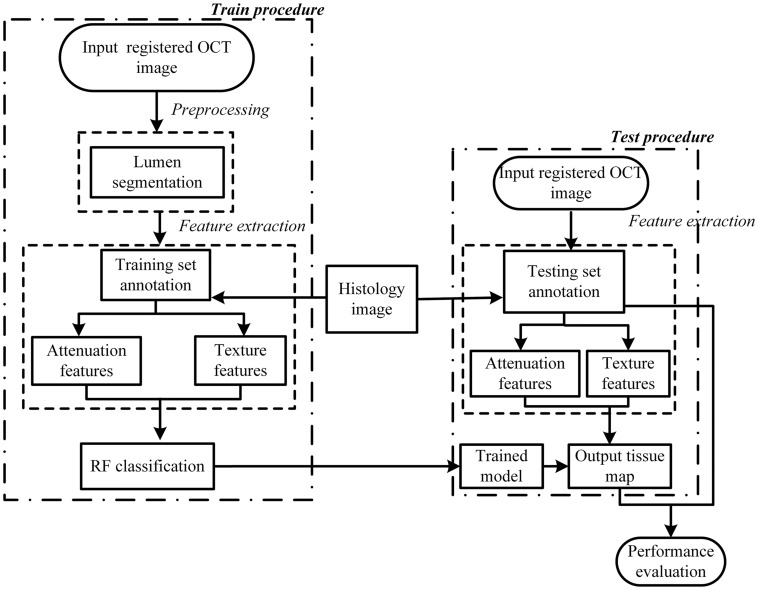
Flowchart of the tissue characterization used in the study.

Figure 2 presents a flowchart of the training, testing procedures and the final performance evaluation. For the training procedure, after the preprocessing steps including automatic guide-wire and lumen segmentation, the optical parameters (OP), the texture features and relation position (RP) features were quantified. Based on these locally extracted values, the supervised pixel-wise classification was applied to train a classifier. The histology slices were used as the gold standard for manually delineating the train and test sets, and an intermediate color-coded image map depicting the different types of tissue was given by the trained classifier. The performance of the algorithm was evaluated by classification accuracy of the trained classifier output and the manual tissue map based on histology.

### Histopathology Analysis

**FIGURE 3:**
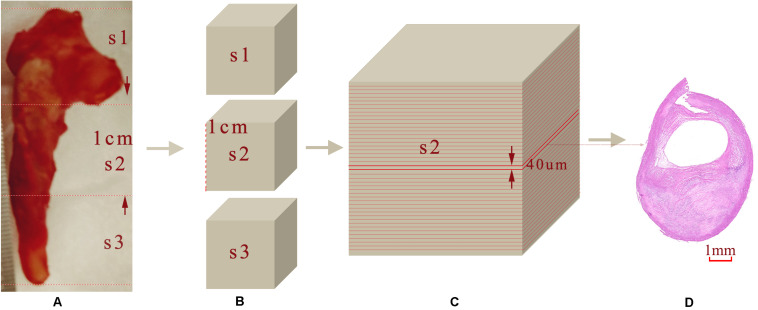
Sample sectioning for histological analysis. **(A)** Carotid plaque tissue. **(B)** Embedding blocks with 1 cm intervals. **(C)** 40 μm interval slices. **(D)** 4 μm histology slices.

After imaging, each carotid plaque tissue was cut into 1 cm serial transverse segments. Each embedded segment was marked in the same order with the corresponding OCT pullback and was serially sectioned into slices of 4 μm transverse sections, with a 40 μm interval perpendicular to the longitudinal axis of the artery. Sections were mounted on the slide glass substrates and then stained with Hematoxylin and Eosin (H&E), Masson’s trichrome, Oil Red O to show the structural and morphology information within the plaque sample. Figure 3 shows the process from the carotid tissue to the slice. The histopathology and immunostained sections were examined with an Axio Lab.A1 (Carl Zeiss, Germany), and the scanned histology images were analyzed using the Pannoramic Viewer image analysis software (Budapest, Hungary). The pathological classification of the plaque components was processed based on the modified American Heart Association (AHA)-classification (Stary et al., 1995).

#### Histopathology and OCT Image Co-registration

The gross morphological features of OCT images such as lumen size and shape, wall size and shape, plaque configuration, as well as calcific plaques were used to adjust the cross-sectional orientation of the slices. Manual co-registration of the OCT images and the digitized histological slices was performed by two experts. The carotid bifurcation and the narrowest lumen (maximum stenosis) were used as the reference points of matching the histology slices and OCT images. The challenge is that the thickness of OCT imaging and histology slices was different. Every 0.1 cm plaque specimen could cover 5 OCT images in one pullback and 250 consecutive histology slices. We were able to use both ends of the plaque samples as additional registration points to co-register the OCT imaging with the histology slices.

#### Ground Truth Annotation

**FIGURE 4:**
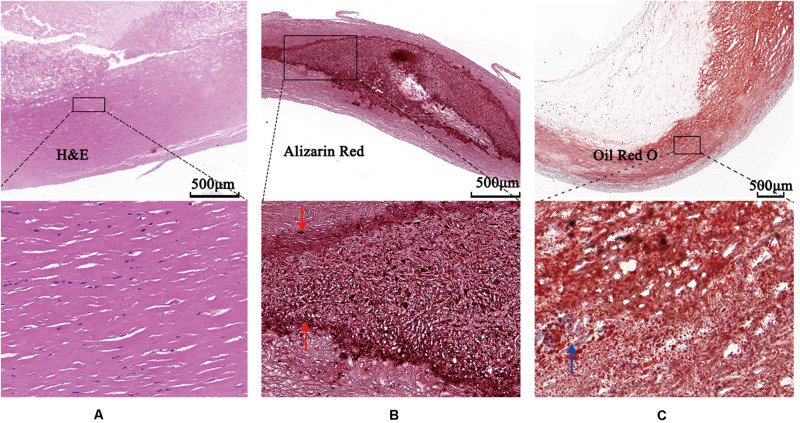
Photomicrographs demonstrating tissue components. **(A)** Low magnification Hematoxylin-Eosin staining reveals fibrous plaque consist of homogenous area that was clearly demarcated by high magnification box (left bottom). **(B)** Low magnification Alizazrin Red staining reveals calcified plaque consist of heterogeneous region that was clear and high-dense internal and external boundaries (middle bottom, high magnification box) (red arrows). **(C)** Low magnification Oil Red O staining shows lipid droplets (blue arrow).

**FIGURE 5:**
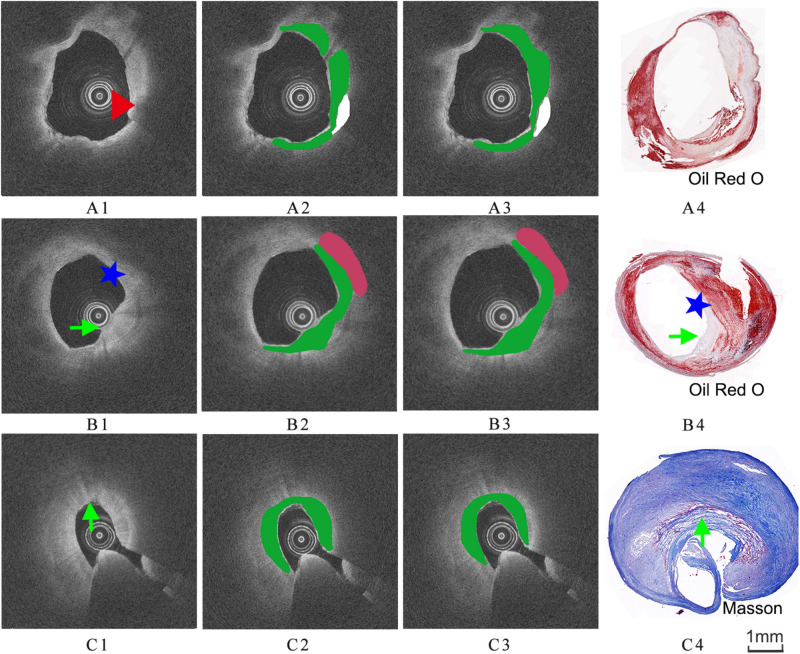
Three groups of co-registration of the histology slices and the OCT images. **(A1,B1,C1)** are cross-sectional OCT image. **(A2,B2,C2)** are the expert 1 annotation OCT images. **(A3,B3,C3)** are the expert 2 annotation OCT images **(A4,B4,C4)** are the corresponding histology slices. Carotid atheroma demonstrates the fibrous region (green arrow), calcification region (red arrowhead) and lipid region (blue asterisk). The plaque morphology and calcified region allowed a precise co-registration between OCT and histology.

The images were annotated by the experts using ImageJ software (Girish and Vijayalakshmi, 2004), according to the registered histology slices. The photomicrograph representative of each tissue type is shown in Figure 4. The two expert independently assessed the plaque components by examining the slice, and outlined the corresponding regions on the OCT image. The three main plaque components were color-coded with a segmentation plugin (Schindelin et al., 2016), such as green for the fibrous tissue, white for the calcified tissue and pink for the lipid tissue. Considering the most important morphologic features of plaque tissue are in the superficial region, whereas it was just within the current OCT imaging capabilities. Therefore, the annotation depth was less than 1 mm from the vessel lumen into the deeper tissue when we defined the main analysis region of the three plaque components. This value is in agreement with the literatures reported in Holzapfel et al. (2005). Figure 5 shows the annotation results by the histology slices.

#### Reproducibility of Annotations

Although the expert performed the annotation, the decision to delineate various tissue map was varying degree subjective and thus prone to analyst variability. To better annotation OCT images, another analyst is necessary to test the reproducibility. To determine reproducibility of annotation results, the intraclass correlation coefficient (ICC) with 95% confidence interval (CI) was calculated to measure the level of agreement between two measurements.

### Pixel-Wise Feature Extraction

#### Inversion Methods for Optical Parameters

OCT systems could acquire multiple OCT A-lines. The intensity of A-line value of the detected OCT signal < *I*_d_(*r*) > can be modeled using a single scattering model according to the Lambert-Beer law (Lantos, 2014).

(1) $$<I_{d}\,\left(r\right)\geq I_{0}\,T\,\left(r\right)\,\hat{s}\,\left(r\right)\,\exp\left(-\mu_{t}\,\left(r\right)\right)$$

(2) $$I_{0}=I^{\prime }\,\mu_{b}$$

where *r* indicates the penetration depth, μ_t_ the total attenuation coefficient and *I*_*0*_ a scale factor, *I*′locally available intensity and μ_b_ the backscattering coefficient (van Soest et al., 2010). The attenuation coefficient μ_t_(*mm*^−1^) is a result of scattering and absorption. However, only scattering can be considered because the contribution of tissue absorption is very low at the near infrared wavelengths used in OCT (Qu et al., 1994). In addition, the OCT signal is influenced by focusing effects related to the confocal properties of the catheter (van Leeuwen et al., 2003), which can be described as:

(3) $$T_{r}={\left[{\left(\frac{r-z_{0}}{z_{R}}\right)}^{2}+1\right]}^{-1\,/\,2}$$

Here *T*_r_ is the longitudinal point spread function (PSF), *z*_*0*_is the position of the beam waist and *z*_R_ is the Rayleigh length. While $\hat{s}$ in (1) represents the modulation due to the OCT depth scan response and is described as follow:

(4) $$\hat{s}\,\left(r\right)=\exp\left[-{\left(\frac{r-z_{C}}{z_{W}}\right)}^{2}\right]$$

While *z*_C_ is the center of the scan, and *z*_W_ is the half width of the roll-off function (Yun et al., 2004).

To reduce the dynamic range and sensitivity to noise of the fitted signal, (1) is linearized by logarithmic transformation:

(5) $$\text{log}\,\left[\left\langle I_{d}\,\left(r\right)\right\rangle \right]-\log\left[T_{r}\right]-\log\left[\hat{s}\,\left(r\right)\right]=\log\left(I_{0}\right)-\mu_{t}\,\left(r\right)$$

The attenuation coefficient μ_t_ and constant parameter *log*⁡(*I*_0_) were calculated using a linear least-square fit to the OCT A-lines for different layers and for different positions of the individual layers by an optimization process.

#### Texture Features

Texture features were extracted from the training set and the testing set images in order to be used for the classification of the plaque tissues. Texture refers to the spatial interrelationships and arrangement of the basic elements of an image. In the study, several texture features were selected as follows:

First order statistics (FOS) textures are directly related to the gray tone distribution of the pixel intensity and ignore inter-pixel correlations. In the paper, four parameters including mean value, standard deviation, skewness and kurtosis were extracted directly from the image. More details can be found in reference (Christodoulou et al., 2003b).

Gray level co-occurrence matrix (GLCM) is a powerful statistical tool for texture analysis, which is a tabulation of how often different combinations of pixel brightness values (gray levels) occur in an image (Soh and Tsatsoulis, 1999). In this paper, the distance was 1 and angle theta were 0°, 45°, 90°, and 135°. Seven features were computed based on the probability density functions (PDFS), including correlation, contract, dissimilarity, energy, entropy, homogeneity, maximum probability.

Neighborhood gray tone difference matrix (NGTDM) corresponds to the visual properties of the texture (Christodoulou et al., 2003a). The following five texture features were extracted form NGTDM, for a neighborhood size of 3 × 3: busyness, contrast, complexity, coarseness and texture length.

Fractal dimension (FD) is an index for characterizing the fractal patterns or sets by quantifying their complexity as a ratio of the change in detail to the change in scale (Soh and Tsatsoulis, 1999). In this paper, the image intensities were transformed to the FD domain using the differential box-counting algorithm (Liu et al., 2003) at various different scales and then displayed for plaque tissue identification.

The optical parameters were one-dimension information extracted by nonlinear fit, the texture feature sets (the four groups) were two-dimension features based on the local neighborhood operations, the sixth set was relative position of pixels (RP) (the *x* and *y* coordinate of each pixel). This RP features were used in combination with other feature sets during the experiment because of their natural and essential characterization. Table 1 presents the details of the feature sets.

**TABLE 1:** Feature sets included in the study.

**Feature sets**	**Fewature name**
OP	μ_t_ and *b*
FOS	Mean, variance, median, skewness, kurtosis
GLCM	Correlation, contract, dissimilarity, energy, entropy, homogeneity, maximum probability
NGTDM	Busyness, contrast, complexity, coarseness, texture length
FD	H^1^, H^2^, H^3^, H^4^
RP	*x* and *y* coordinate

*OP, optical parameters (van Soest et al., 2010), are two paramenters of fit functions. FOS, first order statistics (Christodoulou et al., 2003b); GLCM, gray level co-occurrence matrix (Soh and Tsatsoulis, 1999); NGTDM, neighborhood gray tone difference matrix (Christodoulou et al., 2003a); FD, fractal dimension (Liu et al., 2003); RP, relative position of pixels.*

### Random Forest Classifier

To handle the large training set, the random forest (RF) algorithm was selected (Breiman, 2001). RF is an ensemble of decision trees that combine a series of weak classifiers (i.e., binary trees) to achieve an accurate classification. In addition, the randomization allows the flexibility to explore a large feature space because it only considers a subset of features in each decision tree. The tuning parameters are *Ntree* (number of trees to grow) and *Mtry* (number of variables randomly sampled at each node). Each decision tree is independently produced and each node is split by the parameter *Mtry*. By growing the forest up to another parameter *Ntree*, the algorithm creates trees that have a high variance and a low bias. RF becomes increasingly popular in similar medical image classification applications because of its computational efficiency for large training data, ability to handle multiclass classification.

### Statistical Analysis

Annotation regions were compared on a pixel-wise basis with the results of the RF classifier. The performances of the RF classification methods were compared based on the following model accuracy measures: sensitivity, specificity, accuracy (ACC) (Maroco et al., 2011). In order to avoid the correlation of the results, the training set contained 24 patients, and the testing set contained other 7 patients. The 10-folds cross-validation strategies were applied to estimate the classification performance of the method. The 50 images were randomly split into 10 subsets, each from random set of 5 images. The final statistical results for characterization of plaques component were then calculated based on each testing subset data. After the cross-validation, mean, standard deviation (SD) and median values were computed from the 10 testing set estimations of overall classification accuracy. All statistical analyses were conducted by using Matlab R2018a and related toolboxes (MathWork^®^, Natick, MA, United States): image processing^TM^ and Parallel Computing^TM^ toolboxes.

## Results

### Reproducibility of OCT Images Annotations

**FIGURE 6:**
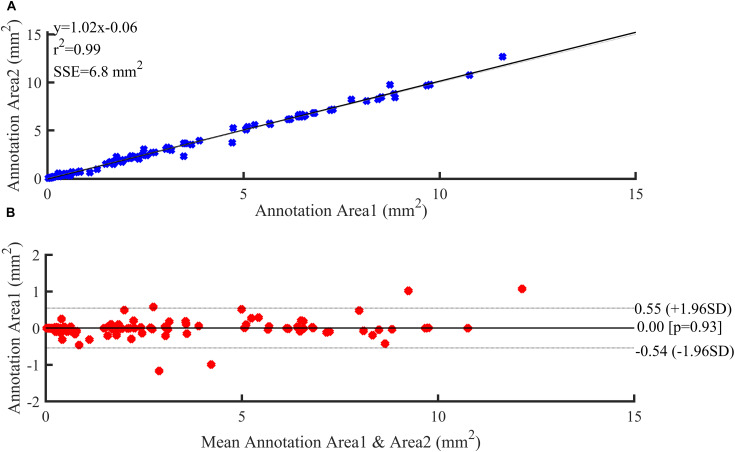
Reproducibility results. **(A)** Regression analysis. **(B)** Bland–Altman plots showing agreement between observers for annotation area.

Figure 6 illustrates the reproducibility results for the two annotations of the three plaque components by the two experts. Both annotation area1 and annotation area2 were in good agreement and displayed a strong linear trend (*R*^2^ = 0.99). The fit lines had slopes of 1.02, and y-intercepts of 0.06 mm^2^ (Figure 6B). A Bland-Altman statistic did not show bias together with narrow limits of agreement (Figure 6A).

### Manual vs Automatic Classification Result

**FIGURE 7:**
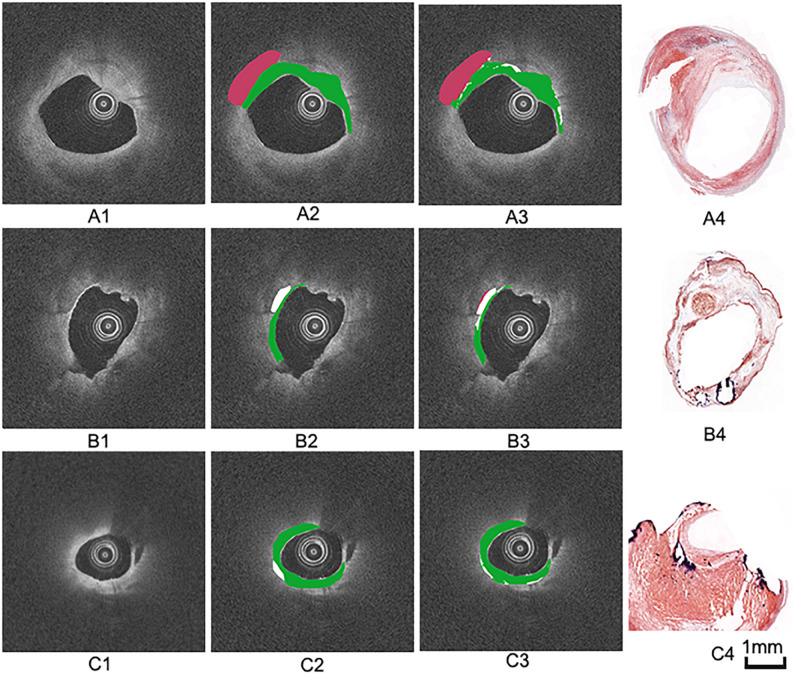
Three groups of manual and automated results. **(A1,B1,C1)** are cross-sectional OCT images from different carotid samples. **(A2,B2,C2)** are corresponding manual annotation results by expert 1. **(A3,B3,C3)** are automated annotation results by the algorithm. Color coding: green = fibrous tissue, white = calcified tissue, red = lipid tissue. **(A4,B4,C4)** are the corresponding histology images.

The annotation results of the two experts show that excellent reproducibility of three plaque components based on the histology slices. Therefore, we choose the annotation result of expert 1 as the benchmark. Figure 7 illustrates examples of the automated classification results compared with histology, respectively, as well as manual annotation.

### Accuracy of Carotid Plaque Classification

Table 2 shows the classification accuracy, sensitivity and specificity of the three plaque components using individual feature set and the combined feature set. The accuracy for the classification of the calcified tissue was lower than those of the fibrous and the lipid tissues. In general, moderate quantitative results were obtained with each individual feature set. The classification results of the three tissue types affect each other, in other words, each type tissue may be misclassified into the other two types. Especially, the fibrous components were often misclassified as the lipid components and vice versa. The highest and lowest classification accuracy of three tissue components were the lipid tissue (83.1%) and the calcified tissue (62.0%) using combination feature, respectively.

**TABLE 2:** Results for each feature set of OCT image compared to the histology.

**Feature sets**		**Fibrous tissue**	**Calcified tissue**	**Lipid tissue**
OP+RP %	Sen	62.7	14.4	66.6
	Spe	59.1	91.5	70.4
	Acc	62.2	13.7	66.4
FOS+RP %	Sen	78.1	41.6	76.9
	Spe	77.0	89.0	87.0
	Acc	77.7	36.0	78.4
GLCM+RP %	Sen	77.5	66.4	81.8
	Spe	92.6	88.0	87.7
	Acc	76.5	67.1	83.8
NGTDM+RP %	Sen	83.1	37.0	74.5
	Spe	76.9	93.4	86.7
	Acc	82.1	35.5	75.7
FD+RP %	Sen	74.1	46.4	83.6
	Spe	83.2	87.3	86.1
	Acc	74.5	48.0	84.6
ALL %	Sen	80.5	64.7	80.2
	Spe	91.2	90.7	87.5
	Acc	80.0	62.0	83.1

### Results of Cross Validation

The results of the 10-folds cross validation are shown in Table 3, which reports mean, standard deviation and median values of the estimates of each classification accuracy. Compared to the fibrous tissue, classification accuracy of calcified and lipid tissue appeared a larger range. The fuse feature set (ALL) obtained the best compromising results between the three plaque tissues.

**TABLE 3:** Result of 10 fold cross validation analysis.

**Feature sets**		**Acc**		
		**Fibrous tissue**	**Calcified tissue**	**Lipid tissue**
OP+RP %	Mean ± SD	62.2 ± 1.6	13.7 ± 4.4	66.4 ± 11.2
	Median	61.7	14.3	66.7
FOS+RP %	Mean ± SD	77.7 ± 1.4	36.0 ± 11.3	78.4 ± 8.7
	Median	77.7	36.1	76.5
FD+RP %	Mean ± SD	74.5 ± 1.4	48.0 ± 7.6	84.6 ± 4.6
	Median	74.6	46.2	81.5
GLCM+RP %	Mean ± SD	76.5 ± 2.7	67.1 ± 4.9	83.7 ± 6.8
	Median	76.5	65.2	83.3
NGTDM+RP %	Mean ± SD	82.2 ± 2.0	3.5 ± 10.1	75.7 ± 8.0
	Median	81.4	31.0	73.9
ALL %	Mean ± SD	80.0 ± 2.1	62.0 ± 8.2	83.1 ± 8.6
	Median	79.6	60.6	81.8

## Discussion

In this paper, we presented an algorithm for the characterization of atherosclerotic plaque components in OCT images and the validation using *ex vivo* carotid plaque tissue (Figure 2).

The present study shows an important finding that the combination of the optical parameters, the texture features and relative position can improve the quantification results, and the validation procedures further facilitated image interpretation. Different methods tend to extract different features, for example, the optical parameters reflect the relationship between the light and the plaque tissue in term of the physical or chemical properties, whereas the texture features reflect the spatial interrelationship of different tissue types. All misclassified tissue types were associated with the other two tissue types (e.g., the calcified tissue was often misclassified as the fibrous and the lipid tissues). The occurrence of the misclassification is mainly due to the blurring of the boundaries of the three main components. Although the literature indicates that the calcified region has a clear boundary, it is difficult to annotate an accurate tissue map in the pixel-wise way. In addition, more feature parameters may be needed to interpret the heterogeneity of OCT tissue components and further distinguish the plaque components.

Recently, CNN have shown remarkable success in medical image processing tasks such as disease classification (Kim et al., 2012), tumor segmentation (Kamnitsas et al., 2017; Harangi, 2018). Abdolmanafi et al. (2017, 2018) used CNN as feature extraction to characterize the layers of coronary arteries and the classification rate was up to 96% of second layer media. Later, they further classified the coronary artery pathological formations (calcification, fibrosis, normal intima, macrophage, media, neovascularization) using CNN as feature extractor, random forest as classifier and majority voting as classification calculation. Gessert et al. (2019) architected the ResNet50-32 and DenseNet-121 network in the different concatenation points and investigated the optimal abstraction level of feature fusion of polar and Cartesian OCT images. The result showed the combined model performed with an accuracy of 91.7%, a sensitivity of 90.9%, and a specificity of 92.4% of the plaque detection in OCT pullbacks. Tissue characterization by OCT images mainly relies on segmentation which is a necessary step for treatment planning in percutaneous coronary intervention (PCI). So far, the studies on the segmentation of plaque components in OCT images using deep learning approaches are very limited. The present study used histology slices as gold standard to annotate the training and testing images, which is a valuable contribution to the interpretation of OCT images and it demonstrates the feasibility of machine learning for plaque components segmentation. This study may provide a foundation for future deep learning-based OCT images classification studies, which will provide a useful tool for the identification of vulnerable plaques and aid the risk stratification of patients with luminal stenosis in the future.

Until now, most research has shown that an atherosclerotic plaque with a lipid or necrotic core and a thin fibrous cap is associated with an increased risk of plaque rupture and thrombus formation, resulting in an acute coronary event or progression of atherosclerosis (Havaei et al., 2017). Therefore, it is important to differentiate the fibrous and the necrotic tissue in order to distinguish a vulnerable and a stable plaque. The developed algorithm is able to characterize the difference between the fibrous and the lipid tissues, which can help in the identification of plaque vulnerability. In addition, fibrous cap rupture and subsequent plaque thrombosis are accompanied with a high macrophage content. Macrophages are inflammatory cells which lead the plaque destabilization by releasing proteolytic enzymes and other pro-inflammatory mediators. Moreover, macrophages tend to scatter light by large organelles (Di Vito et al., 2015). This leads to either a high attenuation coefficient or a high backscatter coefficient of OCT images. Therefore, a large lipid core is often accompanied by a large number of macrophages. Although some literature has shown that OCT is capable of identifying macrophages, the separation of the lipid tissue from the macrophage infiltration is still challenging. In other words, it is difficult to distinguish “poor signal” region in OCT images whether led by macrophage aggregation or infiltration or lipid tissue. In future studies, more data (histology sections and OCT images) may help to better classify the lipid tissues, inflammation regions *etc*.

Although this study is based on *ex vivo* CEA plaque samples, the OCT imaging of the various *ex vivo* plaque components is in agreement with those reported for *in vivo* studies (Mathews et al., 2011; Blackham et al., 2015). The effectiveness and efficiency of intravascular OCT imaging device were confirmed to be at least as good as the imaging information obtained from the *ex vivo* tissues and the OCT imaging was highly reproducible. In addition, the reproducibility of the imaging findings was obtained from arterial segments in patients and animals were identical in both *in vivo* and *ex vivo* images (Mathews et al., 2011). Therefore, it is possible to extend this study to the coronary arteries.

Despite the encouraging results, some limitations still remain. First, it was difficult to co-register OCT with histology because of the inherent differences in the longitudinal resolution between OCT (200 μm) and histology (4 μm). The limitation can be overcome by undertaking continuous histological slide preparation of vessel segments. Second, the data set annotation was performed according to the plaque components and shape of histology image using manual analysis by expert image readers as the ground truth. It is well known that the manual analysis of OCT images tends to cause inter-observer variability and intra-observer variability, and thus resulting in a relatively large deviation. Despite this, it is important to note that some studies have shown the influence of intra-observer on manual image analysis was scarce (Kini et al., 2017). Finally, this study is a single-center study with a relatively small study population. Future multicenter studies or a large amount of histological data would be required to fully test the developed algorithm before it can be used in clinical applications.

## Conclusion

In this study, the combination of the optical parameters and the texture features of OCT images were extracted and used for characterization of carotid atherosclerotic tissue types. The algorithm was validated against histology slices, which were the “gold standard” as the evaluation criteria. This study shows that the developed approach can provide an effective tool for OCT-based plaque vulnerability assessment. Although the statistical results still need to be further improved before the computer-aided automatic segmentation method is applied in routine clinical practice, the fundamental research filled the gap in the quantification and characterization of atherosclerotic tissue types from OCT imaging.

## Data Availability Statement

The datasets generated for this study are available on request to the corresponding author.

## Ethics Statement

The studies involving human participants were reviewed and approved by IEC for Clinical Research of Zhongda Hospital, Affiliated to Southeast University. The patients/participants provided their written informed consent to participate in this study.

## Author Contributions

CH and ZhiL presented the concept and design of the work. CH performed lumen segmentation, the texture feature extraction and RF computations and drafted the manuscript. YH performed the optical parameters extraction. CH, JW, and YY analyzed the data. JW, ZhoL, and ZhiL provided suggestion and editing assistance. ZhiL critically revised the manuscript. All the authors approved the final version and made substantial contributions to this work.

## Conflict of Interest

The authors declare that the research was conducted in the absence of any commercial or financial relationships that could be construed as a potential conflict of interest.
